# Increasing dependability of caregiver implementation fidelity estimates in early intervention: A generalizability and decision study

**DOI:** 10.1177/13623613251374957

**Published:** 2025-10-09

**Authors:** Lauren H Hampton, Micheal P Sandbank, Jerrica Butler, Annabel Garza

**Affiliations:** 1 The University of Texas at Austin, USA; 2 The University of North Carolina at Chapel Hill, USA

**Keywords:** autism, caregiver-implemented interventions, generalizability, Naturalistic Developmental Behavioral Interventions

## Abstract

There is an increasing need to measure caregiver implementation of strategies from Naturalistic Developmental Behavioral Interventions (NDBIs) as a possible key mediator of outcomes in a child. The NDBI Fidelity (NDBI-Fi) rating scheme is a macro-code to estimate the implementation of core strategies. Yet, there is a need to understand the dependability of this measure to ensure intervention study findings are generalizable to everyday interactions and comparable across studies. We addressed this by evaluating the dependability or consistency of NDBI-Fi scores for 20 caregivers, averaged across observations of two occasions of two routines that were each scored by two raters. Our findings indicated that a single score (i.e. from a single occasion, single routine, and single rater) from the measure has low dependability (*g* = 0.43). When scores were averaged across two observations each of two routines scored by two raters (i.e. eight scores total), the score was more dependable (*g* = 0.77). The majority (81.6%) of absolute error variance was attributable to occasions of observation and its interaction with other facets (routine or rater). Therefore, we recommend the NDBI-Fi be applied to more than one observation of more than one routine to strengthen confidence that scores are generalizable to everyday parent-child interactions.

**Lay abstract:**

Outcomes from caregiver-mediated interventions typically include measuring the caregiver’s use of key techniques. The Naturalistic Developmental Behavioral Intervention-Fidelity (NDBI-Fi) tool is a valid measurement strategy for estimating caregiver use. In this study, we sought to understand how to improve data collection from natural observations of caregivers with their children to ensure the scores are representative of how the caregiver and child typically interact. We observed 20 caregiver-child pairs via telehealth in snack and play routines over two different days. Each video was rated using the NDBI-Fi by two observers. We learned that increasing the number of observations may be the best way to improve the dependability of scores from natural caregiver-child observations. This study adds to recent research seeking to understand how to best measure caregiver strategy use. These findings may guide future researchers and clinicians to consider increasing the number of observations used to evaluate caregiver use of intervention techniques in research studies or clinical practice.

Naturalistic Developmental Behavioral Interventions (NDBIs) have demonstrated some of the most dependable, consistent, and effective outcomes for young autistic children (Sandbank et al., 2020a, 2023), particularly in the domains of communication and language (Hampton & Kaiser, 2016; Sandbank et al., 2020b). NDBIs are a class of interventions that share common features, including strategies rooted in behavior analysis as well as in developmental theory (Schreibman et al., 2015). Caregiver-implemented NDBIs rely in part or entirely on the mediation of such strategies through caregiver interaction. That is, clinicians coach children’s caregivers to implement the components of a given NDBI through different modes of instruction.

This model relies on a cascading effect of intervention where the clinician’s implementation of the caregiver coaching directly improves the quality, frequency, and accuracy of the caregiver’s implementation, and the caregiver’s implementation of the NDBI strategies further affects a child’s language, communication, and developmental outcomes by way of increasing interactive learning opportunities. The extent to which the caregiver implements the core NDBI strategies, therefore, has a significant and important impact on the quality and dosage of the intervention that the child receives, and this, in turn, has an important impact on the outcomes in a child. This mediated effect of treatment outcomes has been demonstrated in high-quality clinical trials using caregiver-implemented NDBIs, demonstrating a clear relationship between the caregivers’ implementation of NDBI strategies and the child’s ultimate language and/or communication outcomes (Edmunds et al., 2024; Watson et al., 2017; Yoder et al., 2021).

Although NDBIs were only recently distinguished as philosophically distinct from traditional behavioral and developmental intervention approaches (Schreibman et al., 2015), they now constitute the dominant approach represented in controlled group studies of early interventions for young children on the autism spectrum (Sandbank et al., 2023). The increasing representation of NDBIs in autism intervention research over the last 10 years has created a need for a measure of strategy implementation that can be used to compare the effects of interventions on caregiver implementation across studies. While some named interventions have corresponding manualized fidelity measures specific to those interventions (e.g. Dawson et al., 2010), the NDBI fidelity measure (NDBI-Fi) has quickly gained popularity due to its intended applicability across all NDBI interventions (Frost et al., 2020). This eight-item measure was developed to capture the implementation of core NDBI strategies across daily routines (e.g. snack time, dressing, hand washing) for any intervention packages classified under the NDBI umbrella. The NDBI-Fi has been validated on a large sample of play-based caregiver-child interaction (CCX) videos from multiple research groups and intervention types (Frost et al., 2020) and has demonstrated inter-rater reliability and convergent validity with micro-coding of caregiver use of core NDBI (Sone et al., 2021). However, the extent to which the measure produces a dependable estimate of the caregiver’s overall strategy implementation has yet to be examined.

Studies evaluating the psychometric properties of the NDBI-Fi have focused on establishing its construct validity, convergent validity, and reliability across raters. Yet, no studies have explored the extent to which NDBI-Fi scores are reliable across variations in other facets of the measurement procedures, such as the routine or occasion. Implementation of core NDBI-Fi strategies may vary from day-to-day (occasions) or from one daily routine (routines) to another based on the parent’s or child’s interest, mood, or energy. If variability across routines and occasions is low, then a single observation may be sufficient to estimate the caregivers’ generalized implementation of strategies day-to-day. However, if variability across routines and occasions is high, then averaging the scores across different contexts may improve score dependability.

Generalizability theory (G theory; Cronbach et al., 1972) offers a framework for examining the extent to which different measurement facets contribute error to NDBI-Fi scores. This approach has previously been used in early childhood research to estimate the dependability of communication measures in young children with developmental delay (Sandbank & Yoder, 2014) and the sequential association between educator and autistic preschooler talk (Ford et al., 2025). Generalizability studies (G studies) allow researchers to parse observed variance into true variance (which reflects true differences between people on the intended measured construct and ideally comprises the majority of observed variance) and error variance (which can alter scores incorrectly and limit our ability to capture true differences between people). Error variance can then be further parsed by each measurement facet to evaluate each facet’s relative contribution to measurement error. For example, researchers may learn that the setting for observing a child’s play behaviors (e.g. home, preschool, the playground) contributes a high amount of variability to scores estimating that construct. To address this, researchers may choose to observe the child across multiple settings and average collected scores to obtain a more representative estimate of the child’s true play behavior. Researchers can then use Decision studies (D studies) to estimate the optimal number and combination of observations across a set of facets needed to yield a stable, generalizable, and dependable measure of the desired outcome.

There is a need to optimize the dependability of NDBI-Fi scores across variations in observational contexts, particularly routines and occasions because theory suggests these measurement facets could feasibly contribute error variance to measures derived from child-caregiver interactions. Still, researchers rarely attend to or verify the consistency of scores across any facets beyond rater. Reliance on scores plagued by measurement error increases the likelihood of Type II errors. Thus, by optimizing methods for acquiring stable, dependable, and representative NDBI-Fi scores, we can reduce the threat of Type II error and increase our confidence in the observed caregiver implementation outcomes of caregiver-implemented NDBIs, as well as the likelihood that we will identify potentially true moderated effects of caregiver-undertaken NDBI implementation on the outcomes in a child. This may improve our knowledge about how, when, and for whom caregiver-implemented NDBIs are most effective.

Given this great need, we embedded additional assessment opportunities into an ongoing clinical trial, in which caregiver-undertaken NDBI implementation was measured using the NDBI-Fi scored by two raters from 10-min baseline CCX across two occasions (Time 1 and Time 2) and two routines (play and snack). Our research questions were as follows: (1) To what extent do common measurement facets (occasion, routine, rater) contribute error variance to NDBI-Fi scores of baseline caregiver-implemented NDBI implementation, and which facets are key drivers of error variance? (2) What is the achieved dependability of a single and a combination of NDBI-Fi scores averaged across each of these facets? (3) What is the optimal number of observations and combination of these facets needed to yield dependable averaged scores of caregiver-implemented NDBI implementation?

## Method

### Participants

The current sample was selected from an ongoing clinical trial (R21DC018908) of primary caregivers and their infant/toddlers who have a high likelihood for developmental language disorder and/or autism because of their status as siblings of autistic children (i.e. high-likelihood siblings or HL-Sibs). We included the first 20 participants from this ongoing clinical trial who contributed a complete dataset for all the measures of interest, given the recommendations by Webb and colleagues for including 20 participants with at least two measures per person in G studies (Shavelson et al., 1989) and because G studies do not tolerate any missing data (EduG, 2012). Primarily English-speaking caregivers were recruited to provide consent to participate in the primary institutional review board–approved clinical trial if they lived in the United States, had a child diagnosed with autism, a younger biological sibling of that child between 11 and 18 months of age, and that younger sibling had no diagnosis of a disability or developmental delay and was not receiving early intervention services. Participants were provided with the technology and materials to participate in the virtual study and were able to keep these materials following participation. In addition, participants who completed all surveys in the primary study received $100 gift cards for survey completion.

Among the HL-Sib participants (Table 1), 55% were male, and the age range of the participants was 11–18 months (M = 14.19, SD = 2.2). HL-Sibs were racially and ethnically diverse (75% of HL-Sibs were from marginalized racial/ethnic backgrounds). HL-Sibs demonstrated a range of developmental, expressive, and social communication skills at study intake (Table 1). While ultimate diagnostic outcomes are still under investigation, 70% of siblings were later referred for a full developmental evaluation at the end of study participation due to signs of developmental delay and/or autism observed in our study.

**Table 1.** Sociodemographic/linguistic characteristics of child participants at baseline.

Child Participants (*N* = 20) *N* (%)		
Child biological sex		
Male	11	(55%)
Female	9	(45%)
Child race/Ethnicity		
White (non-Hispanic/Latino)	5 (25%)	
Black	5 (25%)	
American Indian or Alaska Native	1 (5%)	
Hispanic or Latino	4 (20%)	
More than one race	4 (20%)	
Asian	1 (5%)	
Sibling multiplex status	5 (25%)	
Referred for a development or language evaluation following study participation	14 (70%)	
Mean (SD)		
Child age in months	14.19 (2.2)	
Developmental quotient, MSEL	93.75 (14.85)	
Expressive language, MSEL T-score	48.80 (9.77)	
Social-communication skills, CSBS	95.15 (11.81)	

CSBS: Communication and Symbolic Behavior Scales (Wetherby et al., 2003), MSEL: Mullen Scales of Early Learning (Mullen, 1995).

In the current study, the sample of caregivers (Table 2) included 18 mothers, 1 father, and 1 grandmother. Caregivers were on average 35.92 years old (SD = 5.3) and were racially and ethnically diverse (70% of caregivers from marginalized racial/ethnic backgrounds). Families in the sample had a wide range of household income levels ($15,000–249,999), and most primary caregivers were college educated (75% with some college education or higher).

**Table 2.** Sociodemographic/linguistic characteristics of caregiver participants at baseline.

	*N* (%)
Caregiver biological sex	
Male	1 (5%)
Female	19 (95%)
Caregiver race/Ethnicity	
White (non-Hispanic/Latino)	6 (30%)
Black	6 (30%)
American Indian or Alaska Native	1 (5%)
Hispanic or Latino	4 (20%)
More than one race	1 (5%)
Asian	2 (10%)
Family composition in household	
Biological mother and father	18 (90%)
Biological mother and partner	1 (5%)
Biological mother only	1 (5%)
Caregiver role/relationship	
Mother	18 (90%)
Father	1 (5%)
Grandmother	1 (5%)
Caregiver highest level of education	
Some High school	1 (5%)
Some college	3 (15%)
College graduate	9 (45%)
Graduate/professional training or above	6 (30%)
Prefer not to answer	1 (5%)
Income level	
$15,000–$49,999	3 (15%)
$50,000–$99,999	5 (25%)
$100,000–$174,999	8 (40%)
$175,000–$249,999	2 (10%)
Prefer not to answer	2 (10%)
Income qualifies as low income	10 (50%)
Employment status	
Employed full time	15 (75%)
Employed part time	1 (5%)
Stay-at-home caregiver	2 (10%)
Prefer not to answer	2 (10%)
Caregiver age, mean (SD)	35.92 (5.30)

Primary caregiver status was self-selected. Income level includes those who are receiving government support as well as those with income less than twice the poverty level.

### Measures

Participants completed surveys, virtually proctored standardized assessments, and virtually proctored observational assessments at study entry. These assessments were completed across two to three virtual assessment appointments within a 5-week window. Caregivers were given 2 weeks to complete all baseline surveys. All assessments were completed by a research-reliable assessor who had experience working with young autistic children and coaching caregivers. Assessors were masked to all group assignment and allocation sequences. Surveys were sent via RedCAP, a secure, online database used to store and distribute survey data.

### Participant characteristics

Caregiver participants were asked to complete a researcher-developed demographic survey to report on their family characteristics. The older siblings’ autism diagnosis was confirmed through a records review. Children were also characterized by the Communication and Symbolic Behavior Scales (CSBS; Wetherby et al., 2003), as proctored by an assessment coach via telehealth. Similarly, caregivers proctored the Mullen Scales of Early Learning (MSEL; Mullen, 1995) with an assessment coach via telehealth. The CSBS and MSEL, both, yield a developmental quotient with a mean of 100 and a standard deviation of 15.

### Caregiver-child interaction

CCX samples were collected during two telehealth appointments proctored by a research assistant within a 1-month window. We selected baseline observations to first understand the stability of the NDBI-Fi when rated using usual parent-child interactions. Caregivers were provided with a tablet with a case and tripod and a standard set of toys appropriate for infant/toddlers including a textured ball, a baby doll, silicone blocks, cut-able food, a picture board book, and a ball drop. We asked caregivers to reserve these toys for the CCX assessment appointments until the study was completed. In addition, we asked caregivers to have two choices of snack foods available and a drink for the child. We provided all caregivers with a handout about the assessment to review before the appointment.

During the CCX appointment, the caregiver was instructed to interact with their child as they normally do. Then they were asked to interact with their HL-Sib during a snack routine for 10 min and a play routine with the standard set of toys for 10 min. Caregivers were asked to minimize distractions (including turning off the television, removing any highly preferred toys, and selecting a space that would have few interruptions during the sample). Caregivers were asked to set up the tripod at a wide angle of the space to ensure a clear view of both the child and the caregiver. The assessor provided the instructions using a script and then turned off their camera during the observation to further remove distractions. The assessor only interrupted the interaction if the caregiver and child were off camera for more than 20 seconds. All interactions were recorded using a Health-Insurance Portability and Accountability Act (HIPAA)-compliant video conferencing software program.

The CCX videos were rated by three study personnel masked to randomization results who had been trained to exact agreement on at least 80% or more of the items on the NDBI-Fi across three consecutive ratings of training videos prior to study ratings. Raters practiced on three initial videos and reached reliability after scoring an average of five videos. Each video in the current study was rated by two of these raters. Each routine (snack and play) and occasion (first and second occasions) was rated independently and in a random order.

Video recordings were rated using the NDBI-Fi checklist. This checklist provides an estimate of the caregiver’s implementation of NDBI strategies within each activity and at each occasion. The scale includes eight items that are specific to core NDBI strategies, and each item is rated on a 5-point Likert-type scale. Scores from the NDBI-Fi were totaled by averaging together each of the items in the scale. Each occasion, routine, and rater yielded separate total average scores. According to the NDBI-Fi manual, raters are considered as in agreement with one another when they score within one point of one another on any given item. Because of this variability, we included two raters for each video to examine the extent to which rater variability contributes to measurement stability.

### Data analysis

All analyses were conducted using EduG version 6 software (EduG, 2012). Estimates of G can range from 0 to 1.0, and a sufficient G estimate may vary between 0.60 and 0.80 or higher depending on the field of study (Bakeman et al., 1997). For the purposes of this study, we selected a moderate G coefficient of 0.75 as the target for sufficient generalizability. Variance for NDBI-Fi total scores was parsed using a fully crossed G study across the facets of the person (i.e. the caregiver), occasion, routine, rater, and their interaction, with a person set as the facet of differentiation (i.e. the source of true variance that we wish to capture) and occasion, routine, and rater set as facets of instrumentation (i.e. potential sources of error variance that we wish to minimize). The facets of person, occasion, and rater were treated as infinite random, which assumes scores in each facet were sampled from an infinite or extremely large universe of all possible levels within that facet (e.g. all possible caregivers, all possible occasions). Treating facets as infinite random produces more conservative estimates than treating them as fixed and is appropriate if the goal of the study is to generalize estimates beyond the actual sample (Shavelson & Webb, 1991). We chose to treat the facet of routine as finite random, setting the universe at 6, because we assumed that the number of broad routines that typically serve as occasions for measuring CCX is finite and because we wished to generalize beyond those represented in the current study (i.e. generalize from snack and play to book, self-care, or favorite activities). In addition, we conducted a G-facets analysis (akin to item analysis in psychometrics) on the facet of routine to determine whether the exclusion of a given level of this facet (i.e. snack or play) would result in increased measurement reliability. G-facet analysis can only logically be applied to instrumentation facets that are finite. Finally, we used G study estimates regarding the relative contribution of error from each facet to inform subsequent D studies, changing the theoretical number of sampled levels in each facet until we identified optimal measurement designs that estimated sufficient score dependability while minimizing measurement burden.

## Results

The scores from the NDBI-Fi were highly variable across participants and observations, indicating a strong motivation to consider a G study to understand the underlying source of variance. Average NDBI-Fi scores from a single rater ranged from 1.67 to 4.89 across observations and settings (M = 3.41, SD = 0.77). A similar variability was observed when additional raters were added to the averages (1.67–4.89, M = 3.38, SD = 0.73). A similar variability was observed between both snack and play contexts (Table 3).

**Table 3.** Descriptive NDBI-Fi results.

Range (mean (SD))	One rater		Two raters	
	One observation	Two observations	One observation	Two observations
Play	1.78–4.67 (3.38 (0.72))	1.78–4.67 (3.49 (0.74))	1.78–4.67 (3.33 (0.72))	1.67–4.67 (3.45 (0.71))
Snack	1.67–4.44 (3.21 (0.88))	1.67–4.89 (3.30 (0.81))	1.67–4.67 (3.24 (0.79))	1.67–4.89 (3.31 (0.74))
Total	1.67–4.67 (3.29 (0.80))	1.67–4.89 (3.41 (0.77))	1.67–4.67 (3.29 (0.77))	1.67–4.89 (3.38 (0.73))

*Note.* The Naturalistic Developmental Behavioral Intervention-Fidelity (NDBI-Fi) rating scale is scored on a scale of 1–5.

### Variance partition

Estimates of relative and absolute error variance attributable to each source are presented in Table 4. The analysis of variance indicated that the total variance attributable to the person facet (i.e. true variance) was 42.6%, which is low and suggests that the majority of observed variance in scores was attributable to measurement error. When absolute error variance was parsed by source, the vast majority was attributable to the facet of occasion and its interaction with other facets, with 12.2% of absolute error variance attributable directly to differences in occasion (meaning that average scores varied by occasion), 42.2% attributable to the interaction between person and occasion (meaning that the ranking of participants varied by the occasion examined), and 23.4% attributable to the interaction of occasion with person and routine (meaning that the ranking of participants varied by routine within occasion). Measurement routine and rater directly contributed minimal error variance (2.5% and 0.7%, respectively, suggesting there was little difference in average scores by routine and rater), but a relatively substantial portion of error variance was attributable to the interaction between routine and person (11.9%, suggesting that the ranking of participants varied somewhat depending on the routine examined).

**Table 4.** Sources of variance for 20 participants × 2 occasions × 2 routines × 2 rater.

Difference variance	Percent of relative error variance	Percent of absolute error variance
*True variance*		
Person	0.255	
*Error variance*		
Occasion		2.5%
Routine		
Rater		0.7%
Person × Occasion	49.9%	42.2%
Person × Routine	14.1%	11.9%
Person × Rater	1.7%	1.5%
Occasion × Routine		0.0%
Occasion × Rater		0.0%
Routine × Rater		0.0%
Person × Occasion × Routine	27.7%	23.4%
Person × Occasion × Rater	2.9%	2.4%
Person × Routine × Rater	2.0%	1.7%
Occasion × Routine × Rater		0.0%
Person × Occasion × Routine × Rater	1.6%	1.4%
Total	100%	100%
Generalizability parameters	Estimate	
Sum of difference variance	0.255	
Standard deviation	0.505	
Relative standard error	0.312	
Absolute standard error	0.339	
Coefficient	Estimate	
Relative G coefficient	0.72	
Absolute G coefficient	0.69	

### NDBI-Fi total score dependability

When averaged across 2 occasions, 2 routines, and 2 raters (i.e. an average of eight total scores), NDBI-Fi total scores achieved a relative G coefficient of 0.72 and an absolute G coefficient of 0.69. The estimated absolute G coefficient for a single score (i.e. from a single occasion, routine, and rater) was 0.43. In addition, a G-facets analysis suggested that dropping the snack routine would increase the absolute G coefficient, although we note this analysis was exploratory. This analysis indicated that NDBI-Fi total scores achieved a relative G coefficient of 0.74 for the play routine alone across two occasions and one rater (an average of two total scores). Adding the second rater (four total scores) only minimally improved the dependability of this estimate (i.e. 0.74–0.77), likely due to the high levels of agreement between raters.

### Optimization

**Figure 1.**
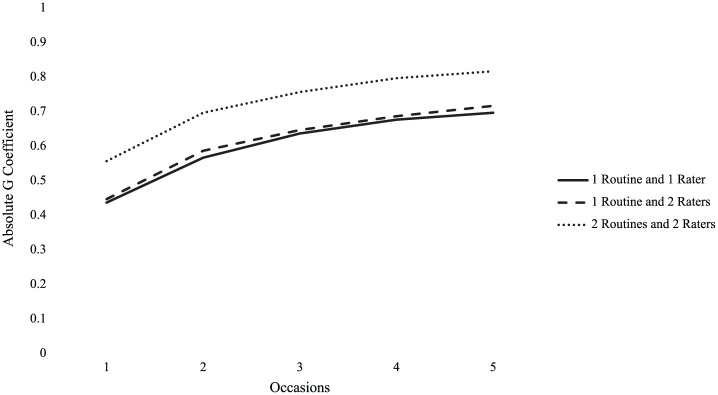
Decision study result estimates of absolute G coefficient across additional occasions for one or two routines across one or two raters.

Measurement optimization estimates are presented in Figure 1. Although the facet of occasion and its interaction with other facets were the largest contributors of error variance, D study results suggest increasing occasions while reducing the number of raters and routines to one each would be an insufficient approach to maximizing score dependability. With only one routine and one rater, an average of scores across five occasions would only achieve an estimated absolute G coefficient of 0.69. Increasing raters to two but keeping routines at one improves on this only minimally, raising the G coefficient achieved with an average of scores across five occasions to 0.71. Averaging scores across two raters, two routines, and five occasions each (i.e. an average of 20 total scores per participant) would produce scores with dependability that exceeded 0.8, but only barely.

**Figure 2.**
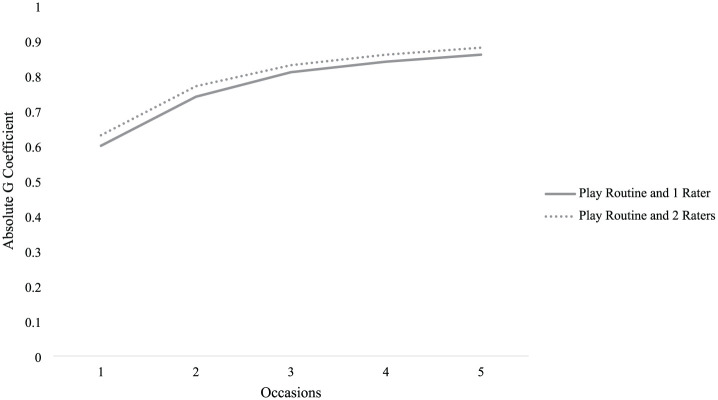
Decision study result estimates of absolute G coefficient across additional occasions for play context alone.

Because the G-facets analysis of the routine facet suggested that the snack measurement routine was associated with less dependable scores and may potentially be an invalid measurement context for this population, we conducted a second D study using only scores from the play routine. Optimization estimates for scores derived only from the play routine are presented in Figure 2. These results suggest that sufficient dependability (i.e. absolute G coefficient > 0.75) can be achieved with an average of scores across two or three occasions (i.e. two or three total scores). Increasing from two occasions to three occasions minimally improves the estimated dependability of this estimate (i.e. from 0.78 to 0.81). Similarly, increasing the number of raters to two for three occasions also only minimally improves the estimated dependability of this estimate (i.e. from 0.81 to 0.83).

## Discussion

Overall, caregiver use of NDBI strategies varied greatly from one occasion to the next (i.e. their Time 1 and Time 2 scores substantially differed) and to a lesser extent from one routine to the next (i.e. their scores for snack and play somewhat differed). Ratings of NDBI strategy use by the caregiver also varied based on the observer, but only minimally. In other words, the occasion of measurement was the key facet driving error variance, and an NDBI-Fi total score derived from a single observation was not a sufficiently dependable measure of caregiver NDBI strategy use. However, we found that low score dependability could be circumvented by averaging scores across measurement facets: averaged scores derived from observations across two occasions and two routines rated by two raters achieved a relatively strong level of dependability (absolute G coefficient = 0.77). Still, to obtain traditionally dependable (e.g. G = 0.8) scores of caregiver NDBI strategy use, we estimated that one would need to average scores from a combination of observations across five occasions and two routines rated by two raters. Given the extent to which the addition of measurement occasions dramatically increases participant burden and research costs, we concluded that the incremental gain in dependability with each added occasion is not sufficient to justify this measurement approach.

We also found limited evidence suggesting that the snack routine may be a less dependable measurement context. If this is the case, removing the snack routine measurement context should increase both NDBI-FI score reliability and validity. Our optimization study using only scores from the play context suggested that if caregiver-child play is the only valid measurement context, sufficient score dependability could be achieved with an average of two or three scores (across two or three occasions). It is important to note that G-facets analysis is based on item analysis, a standard procedure in psychometrics, but makes certain assumptions about the nature of the analyzed facets that may not be completely true. G-facets analysis works only if there are a small finite set of allowable and putatively valid levels of a given facet, akin to test items, rather than a large universe to which the handful of sampled levels is meant to generalize. In conducting the G-facets analysis, we assumed that the routine facet was meant to represent a small finite set of measurement conditions that would be used to derive estimates of NDBI caregiver strategy implementation in research. If the set of potentially valid measurement routines is much larger, G-facets analysis makes little sense. Thus, we urge caution in reliance on the results from the G-facets analysis, as well as those of the subsequent D study restricted to scores from the play routine.

### Recommendations

Given the instability observed from any one application of the NDBI-Fi for a single observation, we recommend that researchers consider collecting more than one observation to estimate caregiver implementation of NDBI strategies. Greater stability is likely to be observed when the same activity is presented; however, this may limit the generalizability of those findings to that specific activity type. Stability may be further improved by averaging across multiple activities, but researchers should be aware that different activities may elicit different applications of the strategies and therefore introduce more instability overall. While these results may have greater generalizability to the caregiver’s implementation throughout the day, it may require more observations to achieve a true stable estimate. Researchers should, therefore, determine the extent to which observing the caregiver in different routines is paramount to the purpose of the observation and measure for a given study. Is the proximal measurement of the caregiver implementing the strategies in a play activity sufficient for the study purpose, or is the caregivers’ generalized use of strategies across daily routines core to the research? Answers to these questions should guide routine selection rather than stability alone.

Alternatively, researchers who have previously collected data with only one observation or activity may consider having two raters complete the NDBI-Fi ratings for all observations to marginally increase stability. Because the NDBI-Fi is rated on a Likert-type scale, it inherently introduces some subjectivity into the rating. This results in a scale for which it is difficult to achieve exact agreement at the item level. Therefore, researchers may slightly improve the dependability of the NDBI-Fi scores by averaging across two (or more) observers’ ratings. While this is not a commonly used approach to improving measurement stability, it is a reasonable and feasible option for those who cannot increase observations of caregiver implementation. In addition, this moves the burden of increasing the stability of the measure from the caregiver (by requiring more observations) back to the research team. While this is a strategy that may increase researcher time and resources, it may still be less time-consuming than other measures of caregiver implementation, such as micro-coding (Dishion et al., 2017). However, levels of agreement between raters in this current study were high, resulting in only minimal improvements in stability (0.74 increased to 0.77) when adding a second rater. Therefore, researchers may weigh the cost-benefit of adding an additional rater.

The findings of this study are likely applicable to the observational context (CCX) in addition to the measure itself. Future research should focus on examining how other macro-codes of semi-structured CCX observations might also replicate the current findings. It is likely that everyday interactions are unstable for any given single interaction, but that similar rates of observations and routines may be necessary for other observational scales of this nature (e.g. Monsi-cc, Vibert et al., 2020; or PICCLO, Innocenti et al., 2023). Similarly, we were unable to examine the extent to which variability was attributable to the child since we observed each caregiver with only one child. However, future research could examine the extent to which caregiver skills are generalizable across children by extending the current study to observations of a caregiver with more than one child. This would allow researchers to understand the extent to which child variability contributes to measurement stability and the extent to which caregivers can generalize their skills to interactions with their other children.

### Limitations

The findings from this study should be interpreted in light of a few important limitations. First, this study used virtual proctored CCX sessions to rate the NDBI-Fi. It is unclear if the results of the current study will generalize to in vivo administrations of the observational probe. Second, some routines that may serve as additional valid contexts for measuring caregiver implementation of NDBI strategies were not used in this study, such as book-reading, sensory play, dressing, handwashing, or play with usual/favorite toys. Further exploration of the facet of routine and the extent to which it may contribute to score dependability is warranted. In these explorations, it is important to distinguish between contexts that are ideal for caregiver use of NDBI strategies and contexts that are ideal for *measuring* caregiver use of NDBI strategies, as these may be overlapping but distinct. For example, researchers may advise caregivers to implement NDBI strategies across all daily routines, but various aspects of some of these routines (such as the structuredness of a book-reading routine or privacy concerns in dressing routines) may limit their utility as contexts for obtaining a representative measure of a caregiver’s generalized strategy use. Third, this study included relatively young infants/toddlers, and therefore, our findings may not readily generalizable to the measurement of caregiver use of NDBI strategies with older children or children with different developmental profiles. While the infant/toddlers in the current study represent a wide range of developmental, communication, and language skills, the current study should be replicated with different populations. Finally, we only explored measurement stability in baseline implementation during this ongoing clinical trial. It is possible, and perhaps likely, that sources of variance may influence stability differently following caregiver instruction on core NDBI strategies such that NDBI strategy use may be more stable following the intervention. The second CCX observation in the current study occurred after a few intervention sessions had occurred for a few participants, but the extent to which intervention influences stability should be explored specifically in a future study.

### Conclusion

The NDBI-Fi is a new tool that is relatively simple to use for measuring caregiver implementation of NDBI strategies. It has face validity for clinical implementation and a high potential for contributing to future knowledge about the extent to which caregivers can implement NDBIs with fidelity, as well as the extent to which caregiver use of NDBI strategies mediates child outcomes. While this study elucidates areas where variability in measurement procedures may reduce the generalizability and dependability of NDBI-FI scores, we hope these results do not dissuade future researchers from using this measure. Rather, the purpose of this article was to highlight the areas where additional consideration around the routines, raters, and number of observations is needed to improve future use and uptake of this measure.

## Supplemental Material

sj-docx-1-aut-10.1177_13623613251374957 – Supplemental material for Increasing dependability of caregiver implementation fidelity estimates in early intervention: A generalizability and decision study Supplemental material, sj-docx-1-aut-10.1177_13623613251374957 for Increasing dependability of caregiver implementation fidelity estimates in early intervention: A generalizability and decision study by Lauren H Hampton, Micheal P Sandbank, Jerrica Butler and Annabel Garza in Autism

## References

[bibr1-13623613251374957] Bakeman R. McArthur D. Quera V. Robinson B. F. (1997). Detecting sequential patterns and determining their reliability with fallible observers. Psychological Methods, 2(4), 357.

[bibr2-13623613251374957] Cronbach L. J. Gleser G. C. Nanda H. Rajaratnam N. (1972). The dependability of measurements. John Wiley & Sons.

[bibr3-13623613251374957] Dawson G. Rogers S. Munson J. Smith M. Winter J. Greenson J. Donaldson A. Varley J. (2010). Randomized, controlled trial of an intervention for toddlers with autism: The Early Start Denver Model. Pediatrics, 125(1), e17–e23. http://doi.org/10.1542/peds.2009-0958 10.1542/peds.2009-0958 PMC4951085 19948568

[bibr4-13623613251374957] Dishion T. J. Mun C. J. Tein J.-Y. Kim H. Shaw D. S. Gardner F. Wilson M. N. Peterson J. (2017). The validation of macro and micro observations of parent–child dynamics using the relationship affect coding system in early childhood. Prevention Science, 18(3), 268–280. 10.1007/s11121-016-0697-5 27620623 PMC5931710

[bibr5-13623613251374957] Edmunds S. R. Hantman R. M. Yoder P. J. Stone W. L. (2024). Parent fidelity mediates the effect of project ImPACT on vocal complexity. Journal of Early Intervention, 46, 174–193. 10.1177/10538151241228461 PMC13367429 42453536

[bibr6-13623613251374957] EduG. (2012). EduG version 6.1-e, generalizability study [Computer software]. Societe Suisse pour la Recherche en Education, Groupe de travail Edumetrie-Qualite de l’evaluation en education. Software prepared by Maurice Dalois and Leo Laroche, Educan Inc, Longueuil, Qc.

[bibr7-13623613251374957] Ford A. L. B. Elmquist M. Johnson L. D. Tapp J. (2025). Preliminary examination of the stability of sequential associations between the talk of educators and autistic preschoolers using generalizability theory. Journal of Speech, Language, and Hearing Research, 68(1), 248–258. 10.1044/2024_JSLHR-24-00195 PMC11842078 39745856

[bibr8-13623613251374957] Frost K. M. Brian J. Gengoux G. W. Hardan A. Rieth S. R. Stahmer A. Ingersoll B. (2020). Identifying and measuring the common elements of naturalistic developmental behavioral interventions for autism spectrum disorder: Development of the NDBI-Fi. Autism, 24, 2285–2297. 32731748 10.1177/1362361320944011 PMC7541530

[bibr9-13623613251374957] Hampton L. H. Kaiser A. P. (2016). Intervention effects on spoken-language outcomes for children with autism: A systematic review and meta-analysis. Journal of Intellectual Disability Research, 60(5), 444–463. 10.1111/jir.12283 27120988

[bibr10-13623613251374957] Innocenti M. S. Vilaseca R. Roggman L. (2023). PICCOLO: observing and coaching caregiver-child interaction to support early development in children with and without disabilities. In Family-Centered Care in Childhood Disability: Theory, Research, Practice (pp. 115–147). Cham: Springer International Publishing.

[bibr11-13623613251374957] Mullen E. (1995). Mullen scales of early learning. American Guidance Services.

[bibr12-13623613251374957] Sandbank M. Bottema-Beutel K. Crowley S. Cassidy M. Dunham K. Feldman J. I. Crank J. Albarran S. A. Raj S. Mahbub P. Woynaroski T. (2020a). Project AIM: Autism intervention meta-analysis for studies of young children. Psychological Bulletin, 146(1), 1–29. 10.1037/bul0000215 31763860 PMC8783568

[bibr13-13623613251374957] Sandbank M. Bottema-Beutel K. Crowley S. Cassidy M. Feldman J. I. Canihuante M. Woynaroski T. (2020b). Intervention effects on language in children with autism: A project AIM meta-analysis. Journal of Speech, Language, and Hearing Research, 63(5), 1537–1560. 10.1044/2020_JSLHR-19-00167 PMC7842122 32384865

[bibr14-13623613251374957] Sandbank M. Bottema-Beutel K. LaPoint S. C. Feldman J. I. Barrett D. J. Caldwell N. Dunham K. Crank J. Albarran S. Woynaroski T. (2023). Autism intervention meta-analysis of early childhood studies (Project AIM): Updated systematic review and secondary analysis. British Medical Journal, 383, Article e076733. https://www.bmj.com/content/383/bmj-2023-076733.short 10.1136/bmj-2023-076733 PMC10644209 37963634

[bibr15-13623613251374957] Sandbank M. Yoder P. (2014). Measuring representative communication in young children with developmental delay. Topics in Early Childhood Special Education, 34(3), 133–141. 10.1177/0271121414528052 25364089 PMC4214868

[bibr16-13623613251374957] Schreibman L. Dawson G. Stahmer A. C. Landa R. Rogers S. J. McGee G. G. Kasari C. Ingersoll B. Kaiser A. P. Bruinsma Y. McNerney E. Wetherby A. Halladay A. (2015). Naturalistic developmental behavioral interventions: Empirically validated treatments for autism spectrum disorder. Journal of Autism and Developmental Disorders, 45, 2411–2428. 10.1007/s10803-015-2407-8 25737021 PMC4513196

[bibr17-13623613251374957] Shavelson R. J. Webb N. M. (1991). Generalizability theory, a primer. Sage.

[bibr18-13623613251374957] Shavelson R. J. Webb N. M. Rowley G. L. (1989). Generalizability theory. American Psychologist, 44(6), 922–932.

[bibr19-13623613251374957] Sone B. J. Kaat A. J. Roberts M. Y. (2021). Measuring parent strategy use in early intervention: Reliability and validity of the Naturalistic Developmental Behavioral Intervention Fidelity Rating Scale across strategy types. Autism, 25(7), 2101–2111. 10.1177/13623613211015003 34030519 PMC8419024

[bibr20-13623613251374957] Vibert B. A. Dufek S. Klein C. B. Choi Y. B. Winter J. Lord C. Kim S. H. (2020). Quantifying caregiver change across early autism interventions using the measure of NDBI strategy implementation: Caregiver change (MONSI-CC). Journal of Autism and Developmental Disorders, 50(4), 1364–1379. 31925669 10.1007/s10803-019-04342-0 PMC7103564

[bibr21-13623613251374957] Watson L. R. Crais E. R. Baranek G. T. Turner-Brown L. Sideris J. Wakeford L. Kinard J. Reznick J. S. Martin K. L. Nowell S. W. (2017). Parent-mediated intervention for one-year-olds screened as at-risk for autism spectrum disorder: A randomized controlled trial. Journal of Autism and Developmental Disorders, 47(11), 3520–3540. 28861651 10.1007/s10803-017-3268-0

[bibr22-13623613251374957] Wetherby A. M. Goldstein H. Cleary J. Allen L. Kublin K. (2003). Early identification of children with communication disorders: Concurrent and predictive validity of the CSBS Developmental Profile. Infants & Young Children, 16(2), 161–174.

[bibr23-13623613251374957] Yoder P. J. Stone W. L. Edmunds S. R. (2021). Parent utilization of ImPACT intervention strategies is a mediator of proximal then distal social communication outcomes in younger siblings of children with ASD. Autism, 25, Article 20946883. 10.1177/1362361320946883 PMC7854804 32811160

