# Supplementary material for: Increasing dependability of caregiver implementation fidelity estimates in early intervention: A generalizability and decision study
Source: Autism. 2025 Oct 9;30(1):187–96. doi: 10.1177/13623613251374957 (PMC12614301; doi:10.1177/13623613251374957)
Supplement: sj-docx-1-aut-10.1177_13623613251374957 – Supplemental material for Increasing dependability of caregiver implementation fidelity estimates in early intervention: A generalizability and decision study [file sj-docx-1-aut-10.1177_13623613251374957.docx]

**Telecoaching CCX Script**

*Ask the following questions to ensure family gets appropriate additional instructions before beginning the CCX.*

1. Do you speak any other languages other than English with CHILD?
   - If yes, include: We ask that you only speak to your child in English during this activity.
2. Are there any other children around at this time?
   - If yes, include: Since this is for research purposes, I ask that you do your best to only play with CHILD during this time. If OTHER CHILD joins in, please redirect them if possible or continue directing the majority of your communication to CHILD. If you need to go check in on OTHER CHILD, feel free to let me know and we can pause the activity.
3. Are there any other adults around at this time?
   - If yes, include: Since we are focusing on CHILD's communication and play with you, we ask that other adults just observe the play session instead of joining in. If you need to check in with ADULT, feel free to let me know and we can pause the activity.
4. This assessment includes a **play portion and a snack portion.** You are welcome to include the snack either at the beginning or the end of the interaction. Both will last about 10 minutes, and usually it is helpful to have snack at the end so we don’t have to end it abruptly. However- this is up to you!
   - *If the child does not eat many foods/snack is aversive….. we can replace this with a sensory activity like playdoh or waterplay. Work with the family to find an appropriate replacement activity. This accommodation should be consistent for the child, and this should not be an option if the child is just not hungry right now.*

**Standard Toys PCX, English**

During this activity we are interested to see how you and CHILD play and communicate with each other while playing with a standard set of toys. During this time, please interact with CHILD just like you normally do. Please also interact with CHILD in the place where you normally do, such as, if you usually play on the floor, at a table, in their room, or in the living room. You can play with any or all of the toys we have dropped off however you'd like. We ask that you avoid playing with any of CHILD's regular toys during this time. This will last for 10 minutes, and I will have my audio and video turned off during this time. I will come back on and tell you when the time is up. I will unmute myself if we need to adjust the camera. Do you have any questions before we begin?

**Snack, English**

During this activity we are interested to see how you and CHILD communicate with each other during a meal or snack. During this time, please interact with CHILD just like you normally do during meals or snacks. You can offer multiple choices or just one- whatever is typical for a snack routine. We ask that you also offer a drink in whatever cup is most familiar to your child. This will last for 10 minutes, and I will have my audio and video turned off during this time. I will come back on and tell you when the time is up. I will also unmute myself if we need to adjust the camera. Do you have any questions before we begin?

*If your child happens to finish snack before the 10 minutes, we ask you to incorporate something that you usually do in the snack routine, such as cleaning up or washing hands, for example. Just so that we can observe the full 10 minutes.

*At the end. The white bag of toys should be cleaned up and stored where your child cannot access them. Please do not allow your child to see or play with these toys at any other time. These are to be saved for assessments only. At the end of the study, you will be able to keep these toys and incorporate them however works best for your family, but for now they should be under restricted access. You will not mail these back to us at any point.*

**OPTIONAL (and only with food aversive children) - Sensory Play Activity, English**

During this activity we are interested to see how you and CHILD communicate with each other during a sensory play activity. During this time, remember for the purposes of this study speak in English and please interact with CHILD just like you normally would during an activity like this. You can offer multiple choices during this sensory play or just one- whatever you feel most comfortable with. If you for any reason need to pause—for another adult or child in your home, please let me know and I can pause the time. This will last for 10 minutes, and I will have my audio and video turned off during this time. I will come back on and tell you when the time is up. I will also unmute myself if we need to adjust the camera. Do you have any questions before we begin?

*At the end. The white bag of toys should be cleaned up and stored where your child cannot access them. Please do not allow your child to see or play with these toys at any other time. These are to be saved for assessments only. At the end of the study, you will be able to keep these toys and incorporate them however works best for your family, but for now they should be under restricted access. You will not mail these back to us at any point.*

***The second caregiver CCX can happen during other standardized assessments, but the CCX with the primary and secondary caregiver should not happen in the same appointment.*

**CCX Troubleshooting**

Although there will be things we cannot foresee and will address in the moment, here are a few things that have occurred in the past and some best practice potential solutions for those. Feel free to continue adding as they come up.

- *If participant video is in an area with low/poor lighting:*
  - Hopefully this is seen before or soon after beginning the assessment but if lighting is an issue, pause and attempt to have caregiver adjust their position.

- - Oftentimes windows and natural lighting can cause the camera to auto-adjust the lighting; things like angling away from windows and more towards people and/or not having a window behind the people (even if shuttered) will help.

- - If worst case you cannot avoid lighting issue, bring the iPad down to floor level with the subjects (but still far enough to get as wide angle as possible). This could be a distractor for child and potentially in reach, so use this as a last resort

- *If participant is out of frame or out of view of camera:*
  - Give participant a few seconds to adjust but if the play/snack seems to continue this way, direct caregiver to adjust as needed. Most often than not, it occurs during play (child has back or profile to camera while in play w/ caregiver) so direct caregiver to move in frame of camera to be seen better **but do not give any cues to orient their bodies due to it potentially skewing scores.**

- - Some participants may be too close to camera and therefore it cuts off toys in foreground or caregiver/child face during assessment. These cases would require more of an adjustment because it would affect visibility across the assessment rather than a brief incident.

- *If child participant is upset/dysregulated:*
  - If this occurs prior to assessment allow caregiver to problem solve (diaper change, Water/food, Need a break? etc.) before beginning. Sometimes tempting child with food items is enough to redirect them to snack or placing toys from CCX bag in reach is enough to redirect to play but pause and allow caregiver time to adjust and give suggestions as needed if it interferes with assessment before continuing.
  - If this occurs during the assessment, ask the parent after 1 minute if they would like to continue or pause. If the child is dysregulated for more than 3 minutes, ask the parent if they would like to continue, pause, or reschedule.
